# Heterogeneity of hard skin layer in wrinkled PDMS surface fabricated by Ar ion-beam irradiation

**DOI:** 10.1038/s41598-018-32378-2

**Published:** 2018-09-19

**Authors:** Seunghun Lee, Eunyeon Byeon, Sunghoon Jung, Do-Geun Kim

**Affiliations:** 1 Department of Physics, Korea Advanced Institute of Science and Technology, Daejeon, 34141 South Korea; 2 Advanced Nano Surface Department, Korea Institute of Materials Science, Changwon, 51510 South Korea

## Abstract

The spatial distribution of binding states in the depth direction of a soft polydimethylsiloxane (PDMS) material was investigated in the hard skin layer formed by Ar ion-beam irradiation. The hard skin layer, typically considered silica-like and homogeneous, was heterogeneous, comprising a topmost layer and an intermediate layer. Impinging Ar ions transferred energy to the PDMS medium by collisional energy transfer, which was maximised at the surface and decreased gradually as the ions penetrated the PDMS. The decreasing energy transfer rate from the surface created a heterogeneous hard skin layer. X-ray photoelectron spectroscopic depth profiling showed the existence of the topmost and intermediate layers. In the topmost layer, scission and cross-linking occurred simultaneously; Si–O bonds showed the dissociated state of SiO_*x*_ (*x* = 1.25–1.5). Under the topmost layer, the intermediate layer showed mostly cross-linking, with Si–O bonds showing silica-like binding states of SiO_*x*_ (*x* = 1.75–2). The spatial distribution of carbon-related bonds such as C–Si and *sp*^3^ C–C also showed heterogeneity, yielding a gradient of bond distribution. A theoretical analysis of the collisional energy transfer rate and displacement per atom showed consistency with the XPS depth profiling results.

## Introduction

Nanostructures of tens to hundreds of nanometers in size on polymer films have various applications such as anti-fouling^1,2^, superhydrophobicity^3,4^, anti-reflection^5^, and surface-enhanced Raman spectroscopy^6^. Such nanostructures on polymers are applied in security^7^ and biotechnology^8,9^. Nanopatterning processes using physical masks or moulds can create nanostructured polymer films with regular patterns, but achieving high-speed and low-cost production is difficult. Demand for the mass production of nanostructured polymer films without physical masks or moulding processes has increased recently^10–16^. To facilitate industrial applications requiring irregularly nanopatterned surfaces, processes using plasma and ion-beam treatment, electrospinning, and electrochemical methods have been used to form nanostructured polymer films; these methods show potential for industrialisation^17^.

Polymer treatment by energetic ions can generate nanostructures directly on polymer films. Ion irradiation of polymer films at several kilo-electron volts induces surface-concentrated polymer deformation by transferring the ion energy locally in a region of tens to hundreds of nanometers^18,19^. The locally delivered energy causes localised bond dissociation and ionisation beneath the surface and initiates scission and cross-linking, which induce self-organised nanostructuring (SONS). In order to design SONS with controlled scission and cross-linking, it is necessary to control the amount of ion energy transferred to the polymers, the depth of energy absorption, and the elements added to change the surface chemical bonds. Ion-beam irradiation is suitable for SONS because it permits easy changes in the incident energy, penetration depth, and reactive ion species, such as oxygen, nitrogen, and hydrogen, for chemical bond modification.

Wrinkles are among the most well-known SONS fabricated by ion-beam irradiation. Wrinkles on polydimethylsiloxane (PDMS) substrates are applicable to wearable devices, as they show excellent flexibility and bio-adaptability^20–23^. Liquid metal ion or gas ion beams can successfully fabricate wrinkles on PDMS substrates^24–26^. X-ray photoelectron spectroscopy (XPS) analysis of wrinkled PDMS surfaces has shown surfaces converted to silica-like hard skin layers. The difference in elastic modulus between the hard skin layer and the PDMS medium induces wrinkling^25,26^. Previous studies have qualitatively attributed the changes in wrinkle structures on PDMS to ion-beam irradiation conditions, such as the ion energy and the incident angle of the beam. Although analyses of the hard skin layer have shown a correlation between wrinkle width and irradiation conditions, a detailed mechanism including physical parameters from ion–polymer interactions has not been reported. Aspects such as the formation of the hard skin layer by collisional energy transfer after ion impingement, the spatial distribution of surface bond states from the variation of ion energy transfer rate with depth, and the distribution of SiO_*x*_ (*x* = 0.5–2) bonds in the silica-like hard skin layer have not been discussed.

In this study, wrinkles were fabricated on a PDMS surface by Ar ion-beam irradiation at the energy of 360–840 eV. XPS depth profiling was used to measure the spatial bond distribution in the depth direction of the wrinkled PDMS. A theoretical program calculated the ion energy transferred to the PDMS in order to describe the correlation between the incident ion energy and the modification depths at which scission and cross-linking occurred. This approach provided a physical parameter to describe quantitatively the formation of the heterogeneous hard skin on PDMS after Ar ion-beam irradiation.

## Results and Discussion

**Figure 1:**
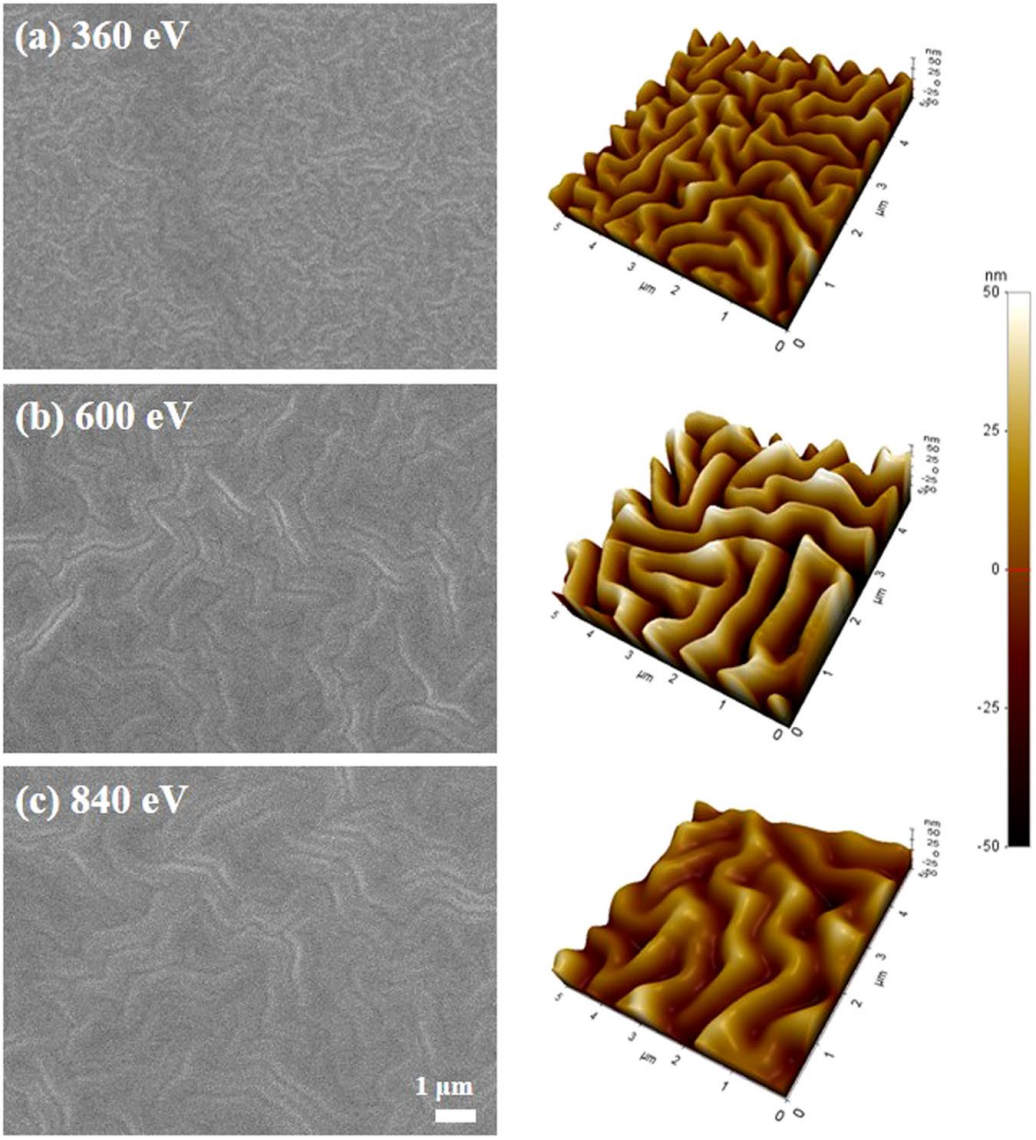
Wrinkled PDMS surface after Ar ion-beam irradiations at different ion energies: (**a**) 360 eV, (**b**) 600 eV, and (**c**) 840 eV.

PDMS samples were prepared by irradiation with Ar ions at the ion energies of 360 eV, 600 eV, and 840 eV. Figure 1 shows the results of scanning electron microscopy (SEM) and atomic force microscopy (AFM) analyses of the Ar ion beam-irradiated PDMS surfaces. As the Ar ion energy is increased, the width of the wrinkles increases from 0.5 μm to 1 μm and the wrinkle height ranges from 20 to 50 nm. This behaviour agrees with other reports of ion beam-irradiated PDMS^25,26^. Transmittance and haze of as-received PDMS sample were 94.52% and 0.28%, respectively. After Ar ion-beam treatment, the transmittance was decreased by 0.22% and haze was increased by 2.18%. The details of optical properties are tabulated in Supplement 1. The PDMS wrinkles produced in this work were fabricated by an in-line ion beam irradiation process using linear ion beams, which yielded uniform ion-beam irradiations for widths reaching 300 mm^27^.

**Figure 2:**
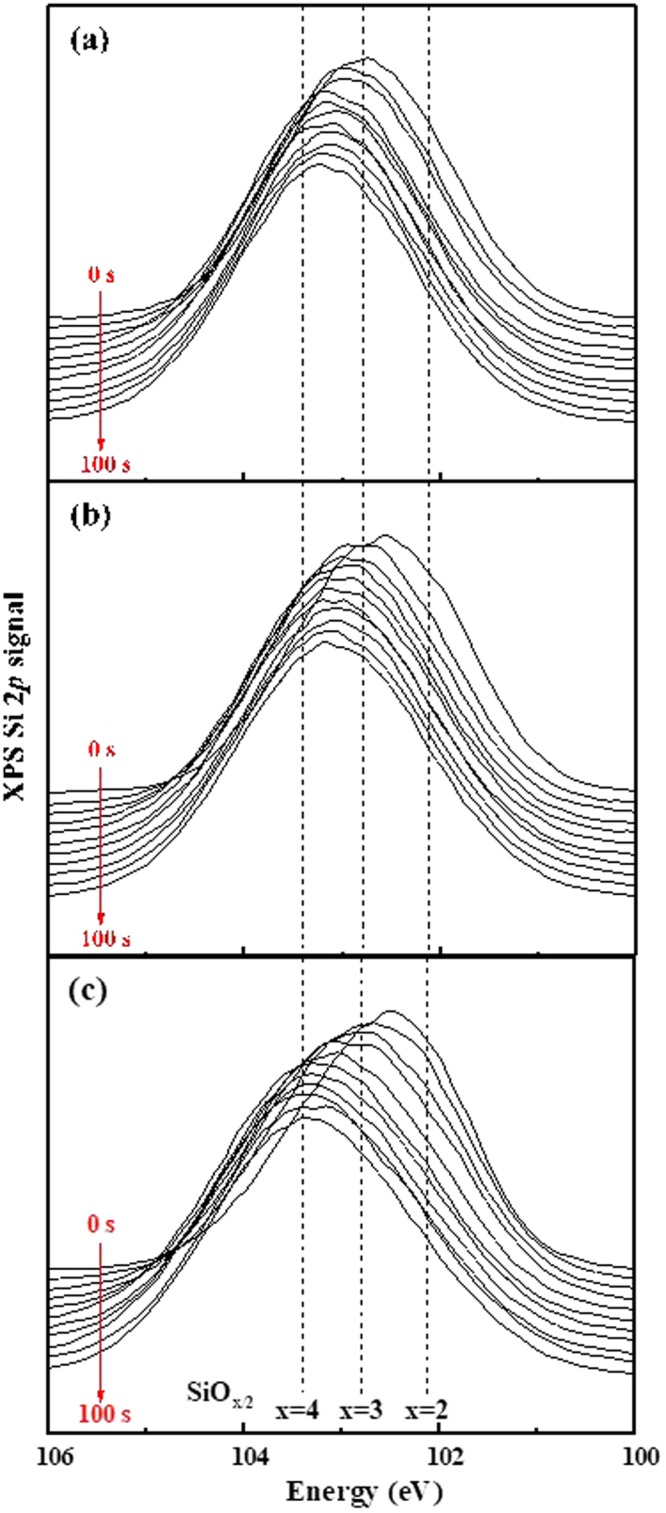
XPS depth profiling of Si 2*p* signal. The etching time is 100 s at intervals of 10 s. Irradiated Ar ion energy: (**a**) 360 eV, (**b**) 600 eV, and (**c**) 840 eV.

XPS depth profiling was performed to analyse the topmost surface of the PDMS irradiated by the Ar ions. The XPS depth profiling of Si 2*p* and C 1 *s* energy levels was conducted in the etching times of 0–100 s at intervals of 10 s. Figure 2(a–c) are the depth profiles of Si 2*p* peak at the Ar ion energies of 360 eV, 600 eV and 840 eV, respectively. The peak positions of SiO_1_ (102.1 eV), SiO_1.5_ (102.8 eV), and SiO_2_ (103.4 eV) bonds are shown in the graph^28^. The peak shift to a high binding energy indicates that the amount of oxygen in the SiO_x_ bond is increased as the etching time increases from 0 to 100 s. At 100 s, bonds similar to SiO_*x*_ (*x* = 1.75–2) are observed at all ion energy conditions. This silica-like layer is the hard skin layer that induces wrinkling^25^. However, in the topmost surface at which the etching time is 0–20 s, the Si 2*p* peak is shifted to a low binding energy, indicating reduced amounts of oxygen in SiO_*x*_. As the Ar ion energy increases to 840 eV, further oxygen reduction occurs in SiO_*x*_ (*x* = 1.5 → 1.25) as shown in Fig. 2(c). Supplement 2 shows the peak deconvolution of Si 2*p* peaks at the etching time of 0 s.

**Figure 3:**
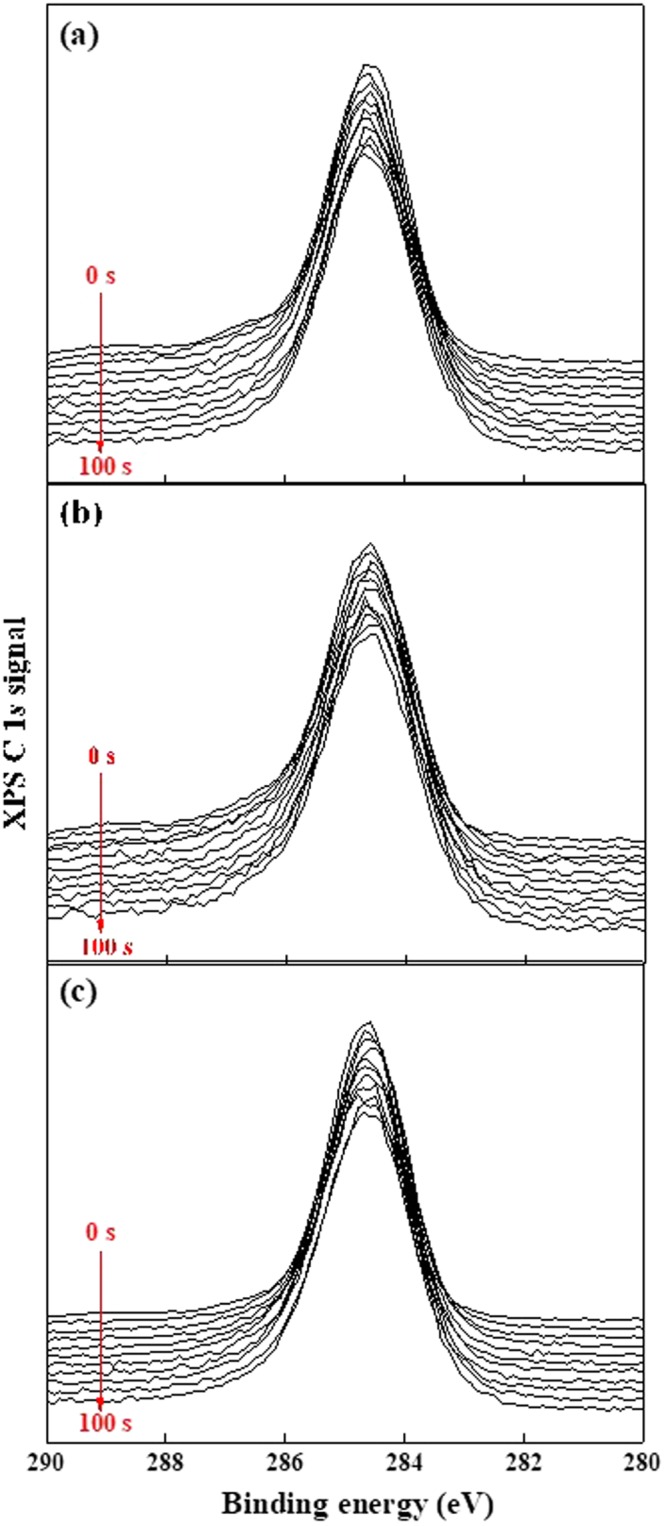
XPS depth profiling of C 1 *s* signal. The etching time is 100 s at intervals of 10 s. Irradiated Ar ion energy: (**a**) 360 eV, (**b**) 600 eV, and (**c**) 840 eV.

**Figure 4:**
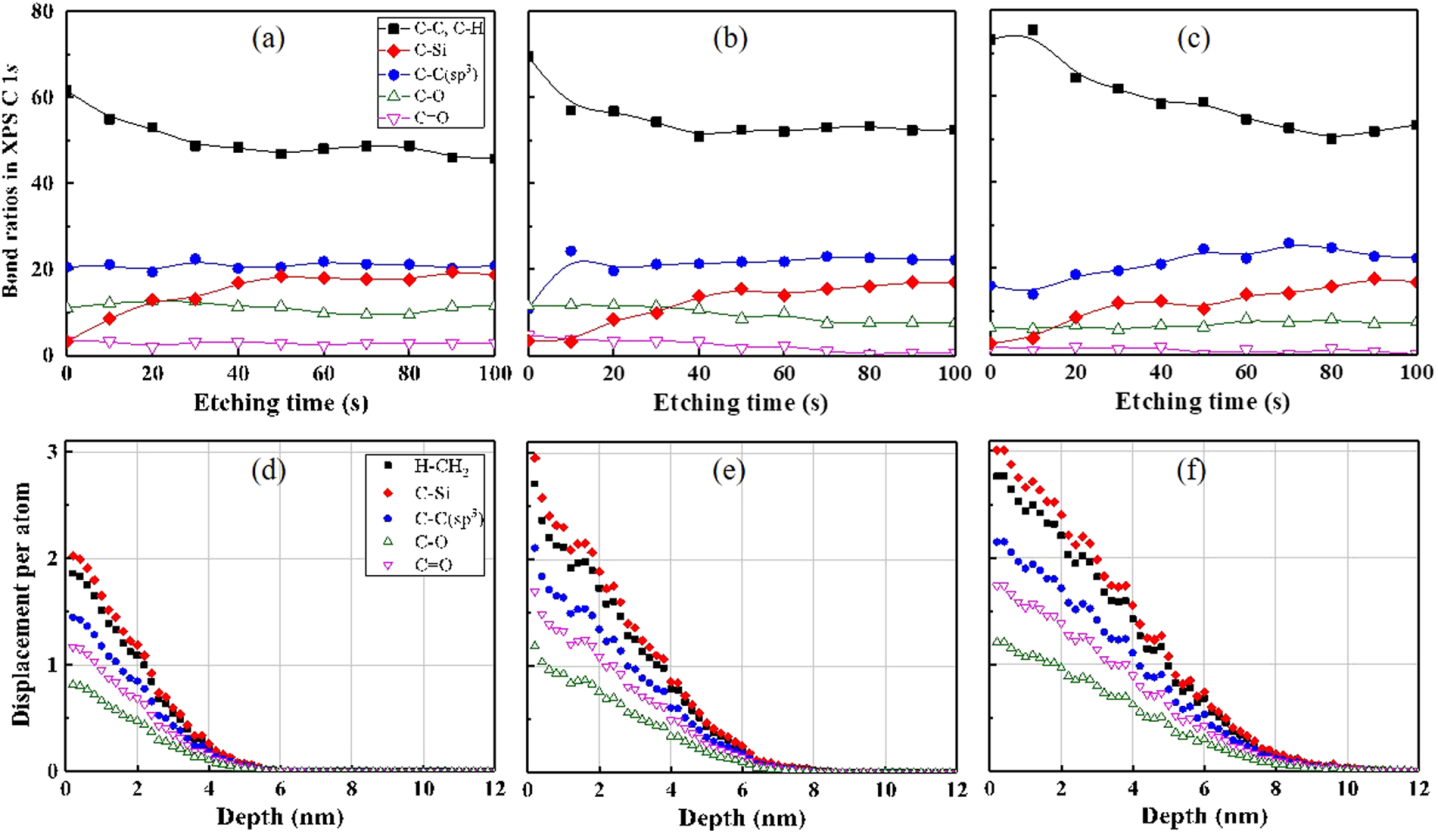
Bond ratio in XPS C 1 *s* depth profiling: (**a**) 360 eV, (**b**) 600 eV, and (**c**) 840 eV; and the DPA with the parameters E_d_ of 4.9, 4.5, 6.3, 7.8, and 11.2 eV for H–CH_2_, C–Si, C–C (*sp*^3^), C=O, and C–O at irradiated Ar ion energy of (**d**) 360 eV, (**e**) 600 eV, and (**f**) 840 eV.

XPS analysis of the C 1 *s* energy level shows more complex but comprehensible results. Figure 3 shows the XPS depth profiling of the C 1 *s* energy level in the etching times of 0–100 s at intervals of 10 s. Figure 3(a–c) are C 1 *s* peaks at the Ar ion energies of 360 eV, 600 eV, and 840 eV, respectively. The XPS C 1 *s* peak includes several bonds, including C–C (*sp*^2^) and C–H overlapped at 284.6 eV, C–C (*sp*^3^) at 285.3 eV, C–Si at 283.8 eV, C–O at 286.3 eV, and C=O at 287.6 eV^29^. The as-received PDMS sample shows a C 1 *s* spectrum including mainly C–H bonds (Supplement 3). Ion-beam irradiation causes cross-linking, inducing the appearance of C–C (*sp*^2^), C–C (*sp*^3^), C–O, and C=O peaks^29^. The increase in the C 1 *s* signal at 284.6 eV after irradiation may arise from C–C (*sp*^2^) formed by cross-linking. This is because ion bombardment dissociates C–H (*E*_*d*_ = 4.5 eV), H–CH (*E*_*d*_ = 4.69 eV), H–CH_2_ (*E*_*d*_ = 4.9 eV) with a higher reaction rate compared to dissociation of the C=O (*E*_*d*_ = 7.8 eV) and C–O (*E*_*d*_ = 11.2 eV) bonds, which require dissociation energies (*E*_*d*_) exceeding those of hydrogen-related bonds^29^. As the etching time increases from 0 to 100 s, the changes of C 1 s peak are observed by peak deconvolution. Figure 4(a–c) show the distributions of C–C (*sp*^2^), C–H, C–C (*sp*^3^), C–Si, C–O, and C=O as functions of depth. Each ratio is calculated by the decomposition area of the C 1 *s* peak (Supplement 3). At the etching time of 100 s, the three samples show similar bond ratios. However, the scission differs for surfaces after etching for 0–50 s. The C–Si bond shows a reduced ratio for short etching times, and the deformed region is extended as the ion energy increases. The C–C (*sp*^3^) bond, which has a higher *E*_*d*_ of 6.3 eV than C–Si (*E*_*d*_ = 4.5 eV), shows scission at the ion energies of 600 eV and 840 eV, but not at 360 eV. The ratios of C=O (*E*_*d*_ = 7.8 eV) and C–O (*E*_*d*_ = 11.2 eV) bonds, which have higher *E*_*d*_ than C–C (*sp*^3^), are almost constant. The increased peak ratio at 284.6 eV is similar to the reduction ratio of C–Si and C–C (*sp*^3^) bonds. This may indicate that the damaged C–Si and C–C (*sp*^3^) bonds are cross-linked to C–C (*sp*^2^).

At the etching time of 150–750 s, there were no significant changes in the XPS signals of Si 2*p* and C 1 *s*, although the XPS depth profile indicates that the Ar ion beam significantly changed the PDMS surfaces compared to bulk PDMS (Supplement 4). The Si 2*p* spectrum indicates that cross-linking reaction forms SiO_*x*_ (*x* = 1.75–2) at the Ar ion energies of 360 eV, 600 eV, and 840 eV. The C 1 *s* depth profile also showed a similar peak at 284.7 eV with broadening, indicating the appearance of cross-linking bonds such as C=O, C–O, and C–C (*sp*^3^).

The heterogeneity of the ion beam-irradiated PDMS surface observed by XPS depth profiling at the etching times of 0–100 s and 150–750 s is understandable via an ion–polymer interaction model based on the collision process. Ions transfer their impinging energy to the polymer medium and generate recoils, which are energetic particles that obtain energy from the ionic collision. In the target medium, the penetrating particles (both ion and recoil) lose energy by nuclear or electronic stopping. When the particle penetrates the polymer, it loses energy by colliding with target nuclei in an interaction called nuclear stopping, which causes atomic displacement that initiates bond breakage and phonon generation^30^. When the energy transferred to the recoil is higher than the dissociation energy or displacement threshold, nuclear stopping induces atomic displacement. If the recoil moves toward the surface and is higher in energy than the surface binding energy, sputtering occurs. For an ion with a transferred energy below the displacement energy, the recoils generate phonons, which release energy over very short lengths of <10 nm. Common polymers are amorphous in phase, and phonons cannot efficiently transfer heat by lattice vibrations. Polymer vibrations transfer heat to the bulk medium at the low thermal conductivity of ~0.1–0.2 W/m·K^31^. This localised heating could induce cross-linking without increasing the bulk temperature^25^. The interaction of the impinging particle with the electrons of the target material can induce electronic stopping, which is inelastic and transfers energy by both electronic excitation and ionisation. All excited electrons (plasmons) eventually lose energy as they thermalise^30^. Electronic stopping effectively induces cross-linking by the collective excited electrons, which produce a large excited volume and therefore compulsive interactions between ions and radical pairs^30^. Thus, the stopping power and the depth at which the target material absorbs energy are critical in explaining the scission and cross-linking reactions occurring at polymer surfaces after ion-beam irradiation.

The concept of the energy loss per unit depth (*dE/dx*) provides a quantitative description of the occurrences of scission and cross-linking in the polymer medium. The d*E*/d*x* is the sum of *(dE/dx)*_*nu*_ for nuclear stopping and *(dE/dx)*_*el*_ for electronic stopping. The *dE/dx* is maximal in the topmost layer, and gradually decreased as the penetration depth increases. While few ions directly lose energy by ionisation and atomic displacement in the topmost layer, the ions transfer most of their energy to recoils. Recoils with energies greater than the ionisation and dissociation energies are concentrated near the topmost layer. Under the topmost layer, electronic stopping causes ionisation, while nuclear stopping mostly induces phonon vibrations. This induces cross-linking in the intermediate layer. Beneath the intermediate layer is the inherent bulk layer. Therefore, the mutual comparison of the dE/dx and the displacement energies of the polymer chains can be used to predict quantitatively the depth-dependent spatial distribution of scission.

In order to explain the heterogeneous hard skin layer, a quantitative value of *dE/dx* is required for varied ion irradiation conditions, such as the ion energy, ion species, and incident angle. Stopping and Range of Ions in Matter (SRIM) is a computational program used to calculate the energy transmitted by ions to a medium through collision^32^. In this study, *dE/dx* is calculated according to the ion-beam irradiation conditions using SRIM. Scission mostly occurs in the topmost layer, where the ion energy transferred to the recoil particle is sufficient to induce atomic displacement. In evaluating scission by atomic displacement, the unit of displacement per atom (DPA) in Eq. (1) is often used^30^:

1 $${\rm{DPA}}=\frac{0.8}{2{E}_{d}}{(\frac{dE}{dx})}_{nu}\times \frac{ion\,fluence}{target\,atomic\,density}$$

**Figure 5:**
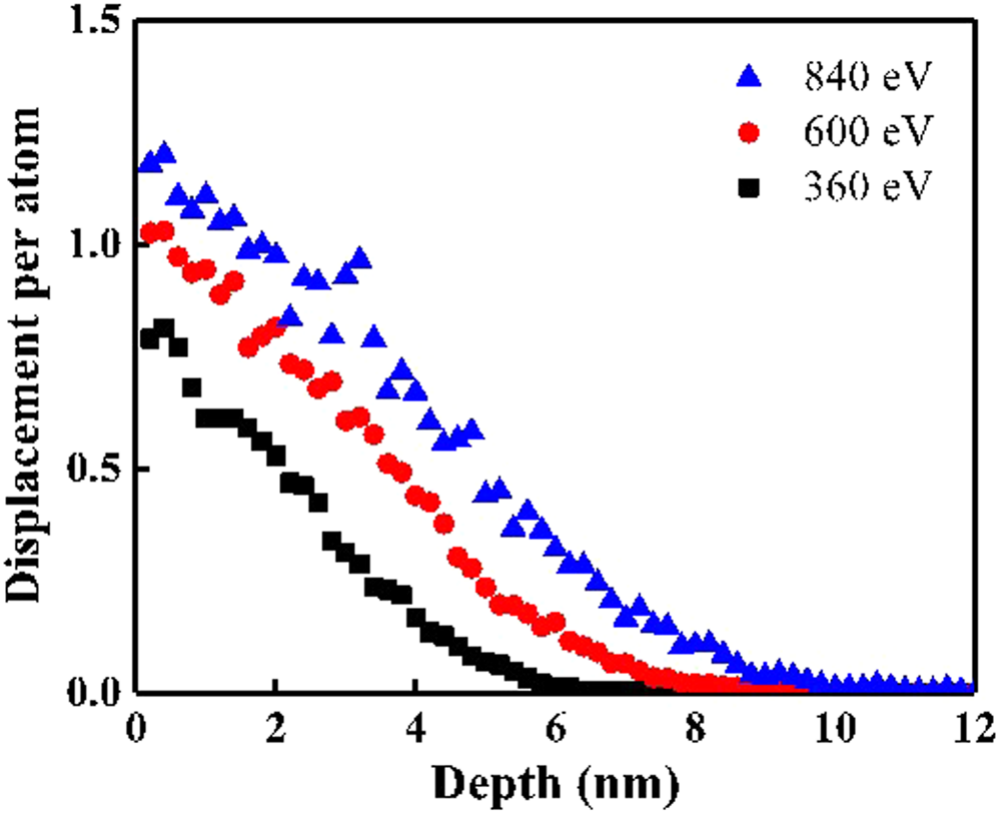
DPA with the parameters of dissociation energy for Si–O dissociation of 8.3 eV.

The DPA is considered a quantitative value describing the spatial scission distribution in the depth direction as a function of *(dE/dx)*_nu_, dissociation energy (*E*_*d*_), and the polymer material. The DPA for the carbon-related bonds were calculated by the parameters including the ion fluence of 5 × 10^15^ cm^−2^, PDMS total atomic density of 0.97 g/cm^3^, and the different *E*_*d*_ of the carbon-related bonds. Figure 4(d–f) show the DPAs for the carbon-related bonds. The low-*E*_*d*_ bonds, such as H–CH_2_ and C–Si, show large DPA values of 1.9–3.0 at the topmost layer where the depth is almost zero. The DPA of C–C (*sp*^3^) is 1.5–2.1, and those of C=O and C–O are lower than 1.8 at the topmost layer. Comparing DPA and XPS depth profiling, scissions of C–Si and C–C (*sp*^3^) occur simultaneously with the cross-linking of C–C (*sp*^2^) and C–H for DPA values higher than 1.9. As the depths at which the DPA is higher than 1.5 increases, the etching times at which the bond ratio of C–Si and C–C (*sp*^3^) is reduced also increase. The DPA for Si–O bonds also showed consistent results with the DPA for carbon-related bonds. The DPA for the atoms of Si–O bonds is calculated by the parameter *E*_*d*_ of 8.3 eV for the Si–O bond. Figure 5 shows that the DPA of the oxygen atom in the Si–O bond is increased from 0.8 to 1.2 at the surface as the Ar ion energy increases from 360 eV to 840 eV. This means that the increasing Ar ion energy induces more O displacements and therefore more Si–O bond scission at the topmost surface. The Si 2*p* peak shift to a low binding energy at the topmost surface in Fig. 2 shows consistency with the increase of DPA for the Si–O bond as the Ar ion energy increases.

**Figure 6:**
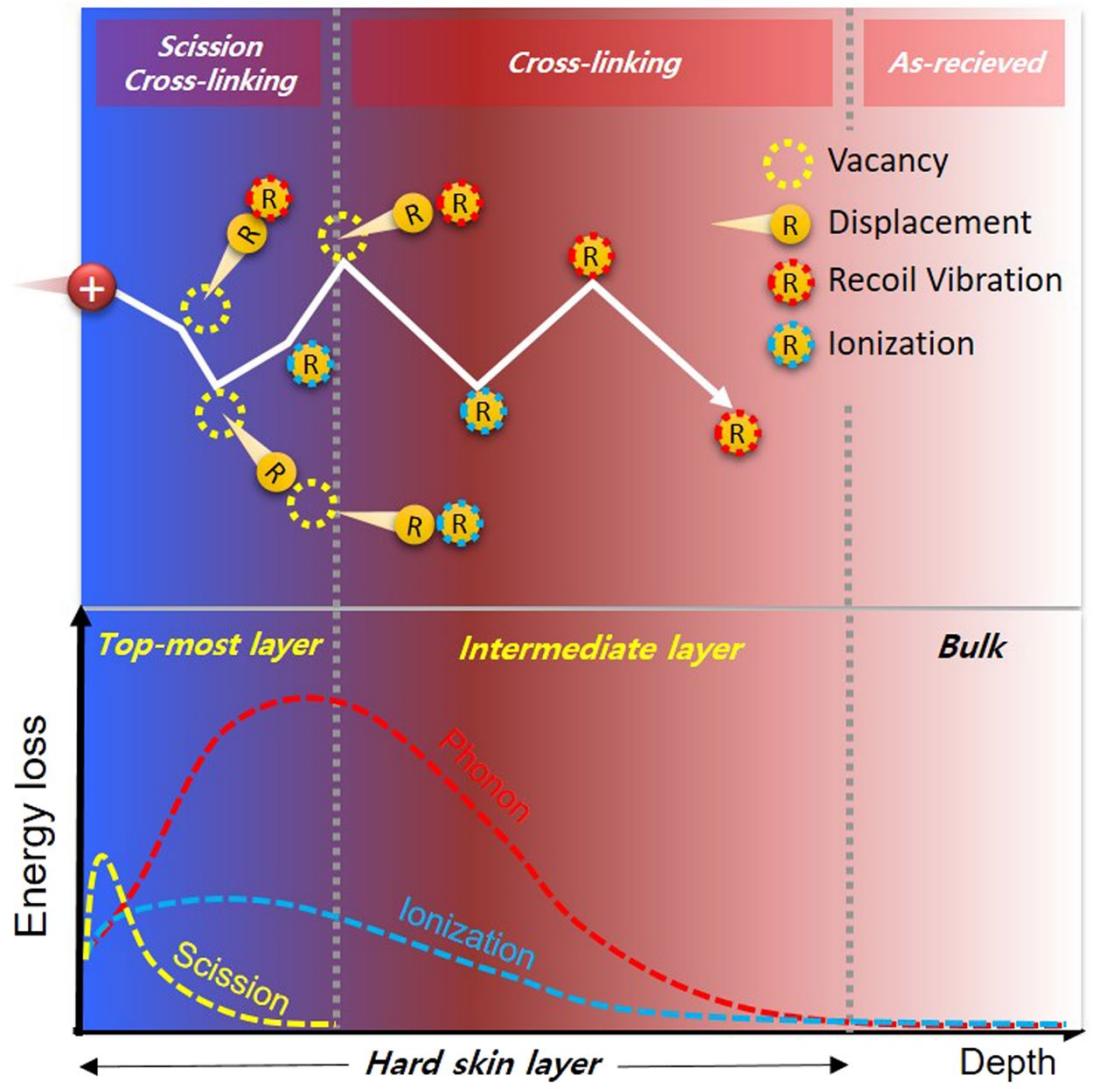
Schematic of the generation mechanism of heterogeneous hard skin layer consisting of topmost and intermediate layers.

In conclusion, the hard skin layer that forms on PDMS treated by Ar ion-beam irradiation has a heterogeneous structure. Energetic stoppings of irradiated ions induce scission and cross-linking simultaneously in the topmost layer. Under the topmost layer, the gradual decrease in stopping power promotes the predominance of cross-linking in the intermediate layer. The overall ion-polymer behaviours inducing the heterogeneity of the hard skin layer are depicted in Fig. 6. The collisional energy transfer rate calculated by SRIM and the concept of DPA allow a quantitative description of the trend in scission in the topmost layer as a function of the Ar ion-beam energy. The comparison between the DPA and XPS depth profiling showed consistency in the spatial distribution in the depth direction of both silicon- and carbon-related bonds. The DPA could be used as a physical parameter to explain scission and cross-linking phenomena initiating SONS on polymer surfaces after ion-beam irradiation.

## Methods

PDMS precursor mixtures were prepared by mixing Sylgard 184 base and hardener (Sylgard 184, Dow Corning Corporation, USA) in the mass ratio of 10:1 in a Petri dish for 10 min. The prepared mixtures were held in a vacuum chamber until no bubbles remained in the bulk of the mixtures. Polyimide substrates of 120 μm in thickness were washed with acetone and IPA in order and dried at 80 °C in an oven for 10 min. The precursor mixtures were spin-coated onto 100 × 100 mm polyimide substrates of 125 μm in thickness for 40 s at 500 rpm. The coated PDMS was cured at 65 °C on a hot plate for 120 min. The PDMS samples on the polyimide substrates were exposed to an Ar ion beam generated by closed-drift ion sources^27^. The ion source emitted a linear Ar ion beam of 300 mm in width and the ion fluence irradiated on the PDMS samples was fixed as 5.0 ± 0.5 × 10^15^ cm^−2^. The anode voltage of the average ion energy was set to 360, 600, and 840 eV. A retarding potential analyser (RPA) measured the average ion energy of the Ar ions irradiated on the PDMS samples^33^. The Ar ion beam irradiation was conducted in a vacuum environment of 0.1 Pa. The surfaces of the wrinkle patterns were observed by field-emission SEM (FE-SEM; JSM-6700F, JEOL) and AFM (NX10, PARK SYSTEMS). The haze and transmittance of the PDMS samples were measured by haze and transmittance measurement system (COH-400, Nippon Denshoku). The surface binding states of the PDMS surface were analysed by XPS (K-ALPHA+ XPS system, Thermo Fisher Scientific). The X-ray source, energy, and spot size were monochromated Al Kα, 72 W, and 400 μm, respectively. The peaks were corrected relative to a C 1 *s* reference (284.6 eV). To minimise changes in the ion beam-treated surface during XPS depth profiling, an Ar cluster gun (energy: 4 kV, cluster number: 1000) was used to etch the PDMS surfaces. The XPS depth profiling was conducted over 100 s at 5 s intervals for the topmost layer, and over 750 s at 30 s intervals for the intermediate layer. In the SRIM simulation, the calculation conditions used are ‘Detailed Calculation with Full Damage Cascades’. This option follows every recoil particle until its energy is less than the lowest energy level of any target atom. To minimise the statistical error of SRIM, the number of collision events exceeds 10000 for each simulation case. The details of the assumptions for SRIM calculation were described in the software manual^32^.

## Electronic supplementary material

supplementary information
