# Author Correction: Heterogeneity of hard skin layer in wrinkled PDMS surface fabricated by Ar ion-beam irradiation

**DOI:** 10.1038/s41598-018-36658-9

**Published:** 2018-12-17

**Authors:** Seunghun Lee, Eunyeon Byeon, Sunghoon Jung, Do-Geun Kim

**Affiliations:** 1 Department of Physics, Korea Advanced Institute of Science and Technology, Daejeon, 34141 South Korea; 2 Advanced Nano Surface Department, Korea Institute of Materials Science, Changwon, 51510 South Korea

Correction to: *Scientific Reports* 10.1038/s41598-018-32378-2, published online 19 September 2018

The original version of this Article omitted an affiliation for Seunghun Lee. The correct affiliations for Seunghun Lee are listed below:

Department of Physics, Korea Advanced Institute of Science and Technology, Daejeon, 34141, South Korea

Advanced Nano Surface Department, Korea Institute of Materials Science, Changwon, 51510, South Korea

This has now been corrected in the HTML and PDF versions of this Article.

