# Supplementary material for: Heterogeneity of hard skin layer in wrinkled PDMS surface fabricated by Ar ion-beam irradiation
Source: Sci Rep. 2018 Sep 19;8:14063. doi: 10.1038/s41598-018-32378-2 (PMC6145931; doi:10.1038/s41598-018-32378-2)

Supplementary information

Heterogeneity of hard skin layer in wrinkled PDMS surface fabricated by Ar ion-beam irradiation

Seunghun Lee*, Eunyeon Byeon, Sunghoon Jeong, Do-Geun Kim

Supplement 1: Optical properties of PDMS samples after Ar ions irradiations.

	Bare PDMS	360 eV	600 eV	840 eV
**Haze**	0.28	0.36	1.00	2.46
**Total Transmittance**	93.52	93.63	92.58	92.30
**Diffusive Transmittance**	0.26	0.34	0.93	2.27
**Parallel Transmittance**	93.26	93.29	91.65	90.03

Supplement 2: Deconvolution of X-ray photoelectron spectra of Si 2*p* binding energy levels at the topmost surface of (a) pristine PDMS and PDMS irradiated by Ar ions with energy of (b) 360 eV, (c) 600 eV, and (d) 840 eV.

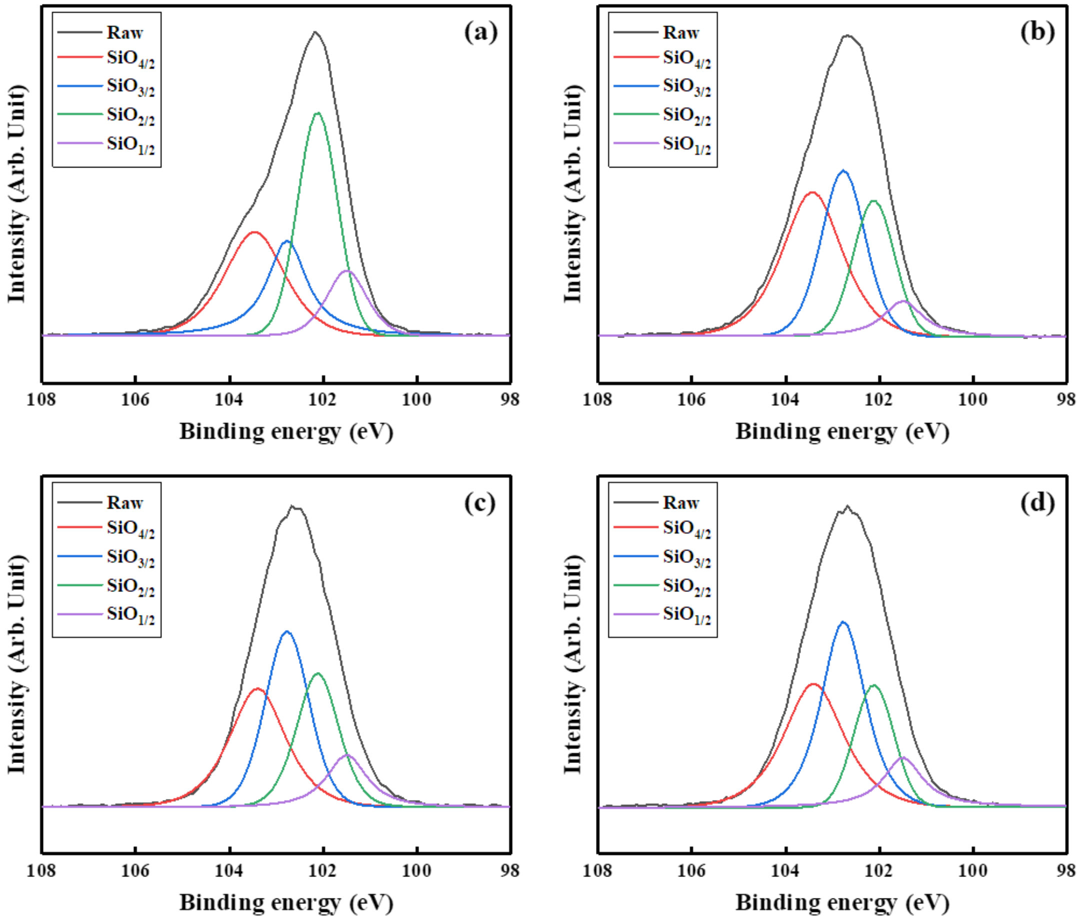

Supplement 3: Deconvolution of X-ray photoelectron spectra of C 1*s* binding energy levels at the topmost surface of (a) pristine PDMS and PDMS irradiated by Ar ions with energy of (b) 360 eV, (c) 600 eV, and (d) 840 eV.

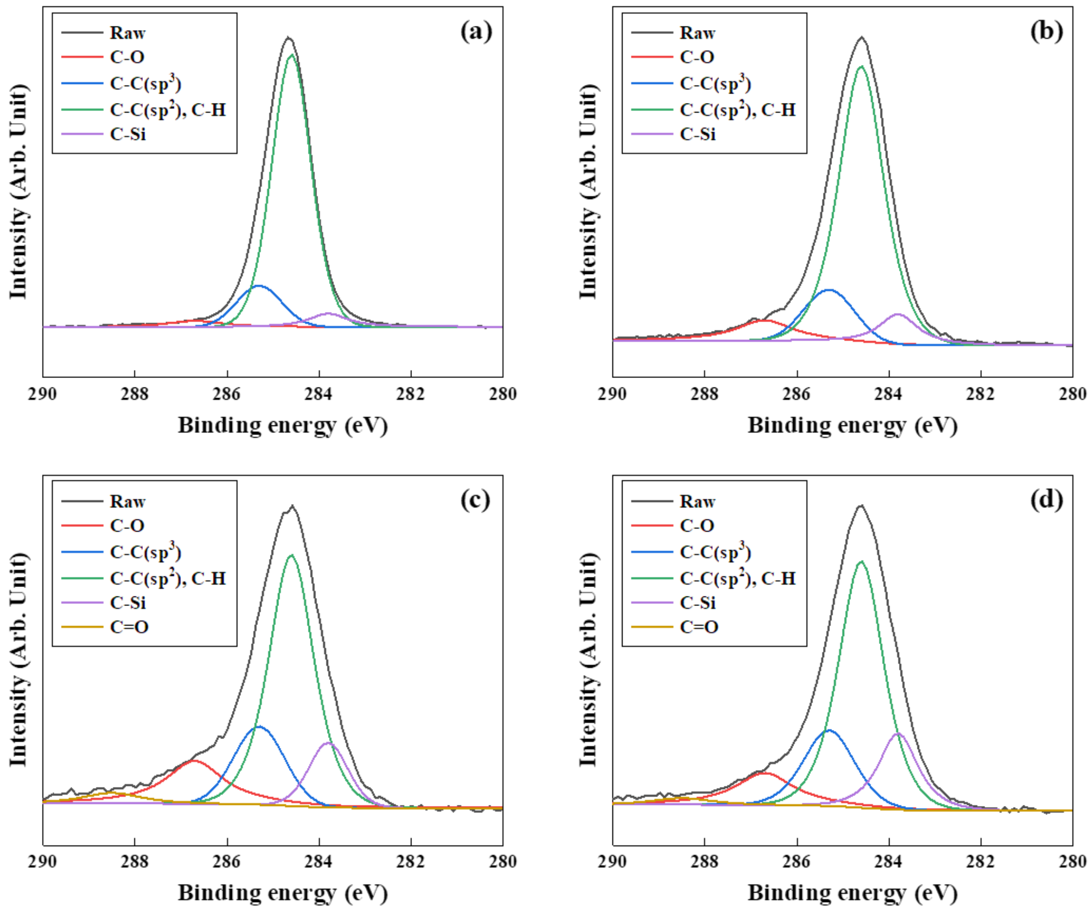

Supplement 4. Bonding states in Si 2*p* and C 1*s* signals at the etching time of 150–750 s when the irradiated Ar ion energy is 300 eV, 600 eV, and 840 eV. (a) Si 2*p* at 360 eV, (b) Si 2*p* at 600 eV, (c) Si 2*p* at 840 eV, (d) C 1*s* at 360 eV, (e) C 1*s* at 600 eV, and (f) C 1*s* at 840 eV. The Si 2*p* and C 1*s* peaks of the as-received PDMS sample are also plotted as a bulk.

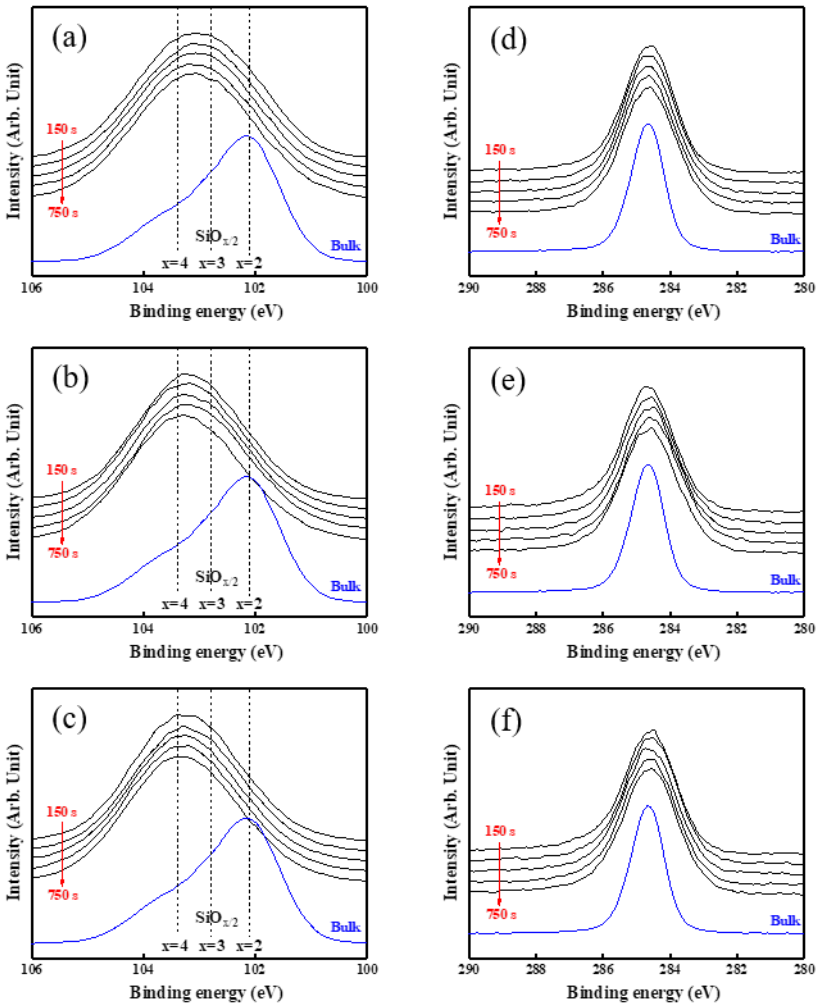

Supplement: Supplementary file 1 — supplementary information [file 41598_2018_32378_MOESM1_ESM.pdf]
